# HELLP Syndrome Complicated with Postpartum Subcapsular Ruptured Liver Hematoma and Purtscher-Like Retinopathy

**DOI:** 10.1155/2012/856135

**Published:** 2012-07-19

**Authors:** Daniela Cernea, Alice Dragoescu, Marius Novac

**Affiliations:** Anesthesiology and Intensive Care Department, County Emergency Hospital, 200516 Craiova, Romania

## Abstract

Purtscher's retinopathy is usually associated with trauma, acute pancreatitis, vasculitis, lupus, and bone fractures. It was rarely described postpartum in patients with preeclampsia as well as associated with HELLP syndrome. We present a case of a multiparous patient aged 44 with severe preeclampsia and postpartum HELLP syndrome complicated with Purtscher-like retinopathy and large ruptured subcapsular liver hematoma that required emergency abdominal surgery after premature delivery of a dead fetus. Postsurgical outcome was favorable regarding both liver function and visual acuity.

## 1. Introduction

HELLP syndrome is a serious complication of preeclampsia characterized by hemolysis, increased liver enzymes, and thrombocytopenia. The most likely cause is the microangiopathy that occurs in pregnancy. HELLP syndrome usually develops before birth, with only one-third occurring postpartum. Risk factors for HELLP syndrome are age above 35 and multiparity [1]. 

The pathogenesis of HELLP syndrome is not yet fully known, but most likely it is related with the microangiopathy developed during pregnancy and the subsequent endothelial dysfunction, with activation of the intravascular coagulation cascade. Hemolysis is caused by vascular injury while hepatic blood flow obstruction due to fibrin deposits in the hepatic sinusoids leads to liver enzyme increase and occasionally formation of subcapsular liver hematoma or even liver rupture [2].

Purtscher's retinopathy is usually associated with trauma, acute pancreatitis, vasculitis, lupus, and bone fractures. It was rarely described postpartum in patients with preeclampsia as well as associated with HELLP syndrome (hemolysis elevated liver enzymes low platelets) [3]. 

## 2. Case Report

We present a case diagnosed with ruptured subcapsular liver hematoma and Purtscher-like retinopathy in a patient with postpartum HELLP syndrome. 

A multiparous (pregnancies 8 and deliveries 3) pregnant woman aged 44 is admitted at 34 weeks of gestation in the emergency room at a city hospital for lack of fetal movements, painful uterine contractions, and headache. Obstetrical examination reveals a dead fetus. The patient presents 180/125 mmHg blood pressure, proteinuria (4 g/dL), and bilateral leg edema and is diagnosed with severe preeclampsia. The patient is given intensive antihypertensive treatment, but blood pressure remains elevated (160/110 mmHg). 

A few hours later, the patient spontaneously gives birth to an 1100 g dead male fetus. Postpartum, despite intensive antihypertensive treatment, blood pressure remains elevated (160/120 mmHg) with a heart rate of 130 beats/min and general pallor. Shortly after fetal expulsion, the patient begins to experience acute pain in the right upper quadrant irradiated towards the right shoulder as well as signs of severe anemia. Lab analysis confirms severe anemia (Hb = 7.63 g/dL, Ht = 23%), elevated liver enzymes (AST = 418 U/l, ALT = 596 U/l), and thrombocytopenia (Plt = 110.000/mm^3^). The patient is sent to a higher degree hospital with the clinical suspicion of HELLP syndrome and hepatic subcapsular hematoma for further investigation and appropriate therapeutic conduct.

Patient's condition worsens, with intense pallor, severe anemia (Hb = 5.3 g/dL, Ht = 16%), decreasing platelets (Plt = 90.000/mm^3^), increased liver enzymes (AST = 578 U/l, ALT = 732 U/l), elevated lactate dehydrogenase (600 U/l), and total bilirubin (BT = 3.5 mg/dL). Clinical examination reveals a distended and painful abdomen, with signs of peritoneal irritation and positive Mandel sign. 

**Figure 1:**
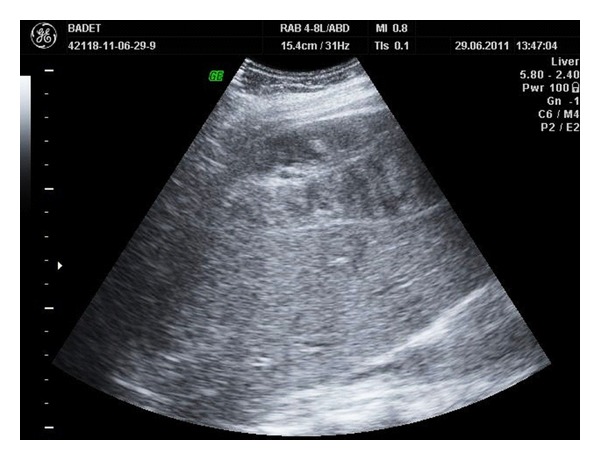
Liver ultrasound reveals large subcapsular hematoma.

**Figure 2:**
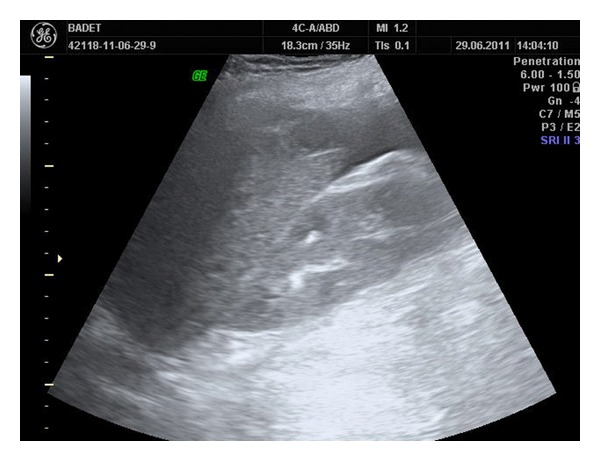
Liver ultrasound after 15 minutes reveals growth of the subcapsular hematoma.

Emergency abdominal ultrasound examination identifies heterogeneous liver structure with a 9/5 cm hypoechoic lesion in the right lobe, suggestive for a subcapsular hematoma and moderate quantity of perihepatic fluid in the Morrison*ʼ*s space and Douglas*ʼ*s pouch (Figure 1). The next examination performed after 15 minutes describes enlargement of the subcapsular hematoma image (12/7 cm) as well as the volume of the Morrison*ʼ*s and Douglas*ʼ*s space fluid (Figure 2).

**Figure 3:**
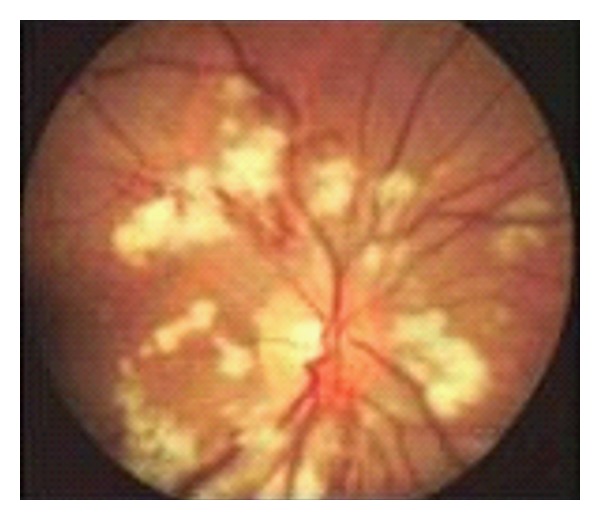
Fundus examination shows characteristic cotton-wool exudates.

Along with the alteration of the patient condition, she accuses loss of visual acuity and, consequently, an eye examination is performed. Eye examination reveals diminished visual acuity in both eyes: 0.05—right eye and 0.1—left eye. Intraocular pressure: 17 mmHg—right eye and 18 mmHg—left eye. Pupillary light reflexes are present bilaterally with a normal anterior pole. Fundus examination describes similar changes bilaterally: papillar edema with blurred papillar contour, various sizes of peripapillar cotton wool exudates along the vascular temporal arcades, mild retinal edema, and normal peripheral retina (Figure 3). The diagnosis is Purtscher-like retinopathy.

Patient's general condition continued to worsen, and she has clinical signs of hemorrhagic shock: dizziness, intense pallor, cold extremities, low blood pressure (80/55 mmHg), sinus tachycardia (HR = 130/min), weak peripheral pulse, and vomiting. Emergency exploratory laparotomy is consequently performed, finding an important amount of blood in the peritoneal cavity (1500 mL) and a large ruptured liver hematoma occupying the entire diaphragmatic surface of the right liver lobe, which is evacuated (1000 mL blood clots) followed by surgical haemostasis with collagen patches and perihepatic drainage associated with intensive fluid replacement, as well as blood and fresh-frozen plasma transfusions. 

Postoperatively, the patient's condition gradually improved, with no further complications. She was discharged after 16 days of follow up. Ultrasound control at discharge showed a normal-sized liver with homogenous structure without pathologic processes. Patient's visual acuity progressively improved during hospitalization (0.2—right eye and 0.3—left eye at discharge), and the eye examination before discharge revealed diminished papillar edema and exudates bilaterally.

## 3. Discussion

HELLP-syndrome-complicated pregnancies can develop severe complications such as disseminated intravascular coagulation, abruptio placentae, acute renal failure, pulmonary edema, and hepatic subcapsular hematoma [4]. Liver hemorrhages and liver ruptures with subsequent bleeding are the most severe complications associated with HELLP syndrome with a mortality rate of 18–86% [5].

Pathogenesis of hepatic subcapsular hematoma associated with HELLP syndrome is not yet fully known. It is assumed to be secondary to fibrin thrombus formation within the liver arterioles and sinusoid capillaries with subsequent periportal necrosis, intrahepatic hemorrhage, subcapsular liver hematoma, or liver rupture. The most commonly affected is the right hepatic lobe [6].

Symptoms of the liver subcapsular hematoma associated with HELLP syndrome are nonspecific and include nausea, vomiting, epigastric or right upper quadrant pain irradiated towards the right shoulder, and signs of hypovolemic shock developing when the hematoma is ruptured and severe abdominal bleeding occurs [7].

Diagnosis of hepatic subcapsular hematoma in patients with HELLP syndrome is supported by symptoms and laboratory and imagistic tests that are required for any suspicion of impaired liver function. Abdominal ultrasound is the faster noninvasive method of diagnosis and evaluation. Serial ultrasound can diagnose the increase in size of a liver hematoma or rupture so that treatment can be instituted quickly and effectively.

Differential diagnosis can be made using abdominal ultrasound, computed tomography, or nuclear magnetic resonance.

Treatment of subcapsular liver hematoma associated with HELLP syndrome should be initiated in specialized centers where a quick diagnosis and optimal treatment can be achieved. Thus, hemodynamic stable patients should be treated conservatively; the therapeutic management includes: intensive fluid replacement, transfusions of blood, fresh-frozen plasma, and/or platelets according to case, with or without percutaneous hepatic artery embolization.

If the hepatic subcapsular hematoma is ruptured and patients are hemodynamic unstable, emergency surgical intervention should be performed with collagen fleece packing of the bleeding surfaces and drainage of the perihepatic space. Uncontrollable liver bleeding leading to acute hepatic failure may require liver transplant [8].

In our case, the patient had clinical signs of hemorrhagic shock, and serial ultrasound images show increased hepatic subcapsular hematoma size and its rupture; so that surgery was the only option to save the life of the patient.

Purtscher's retinopathy was first described in 1910 by O. Purtscher in a patient with severe craniocerebral trauma. Later it was described as the retinopathy associated with other pathologies such as acute pancreatitis, chest trauma, long-bone fractures, or postoperative, in orthopedic surgery, lymphoproliferative disease, after Valsalva maneuver and retrobulbar anesthesia.

Purtscher-like retinopathy occurrence in patients with preeclampsia and HELLP syndrome was very rarely described. Clinical manifestations are typically bilateral. Decreased visual acuity range is usually at 20/200 and counting of fingers. Recovery usually takes several months depending on the severity of retinal damage. Several studies have shown that in more than half of the cases of Purtscher-like retinopathy associated with preeclampsia recovery of retinal lesions was not complete [9]. In our case, the recovery was only partial at discharge after 16 days with decreased visual acuity, which gradually improved during the following months.

Pathogenesis of Purtscher retinopathy is determined by the retinal arteriolar embolization by leukocyte aggregation in response to complement activation. The cause of Purtscher-like retinopathy associated with HELLP syndrome is not fully known, with the assumption being that exposure to amniotic fluid causes C5a-activated leukocyte embolization. 

The onset of HELLP syndrome is believed to be triggered by the same factors as preeclampsia, namely, endothelial dysfunction, inflammation, complement activation, platelets consumption, and neutrophilia. Endothelial injury causes fibrin deposits formation, platelet aggregation, and hemolysis, while chronic inflammation of the deciduous placenta leads to complement activation and complement 5a binding to the endothelial cells which subsequently induce neutrophils aggregation and leukocyte embolization. This theory explains the appearance of Purtscher-like retinopathy in preeclampsia and HELLP syndrome [3]. 

Purtscher-like retinopathy is characterized by sudden decrease in visual acuity [10], associated with the following aspects of the fundus: papillar edema, ischemia of the posterior pole in one or both eyes, optic disk swelling, cotton wool exudates, and sometimes hemorrhages around the papilla and the back pole. Purtscher flecken described as multiple areas of polygonal retinal whitening between the retinal arterioles and venules usually accompanies the superficial cotton-wool spots at the lower pole [11]. Our patient had papillar edema with blurred papillar contour, various sizes of peripapillar cotton wool exudates along the vascular temporal arcades and mild retinian edema. No hemorrhages and Purtscher flecken were described in this case.

## 4. Conclusion

This paper shows that preeclampsia followed by HELLP syndrome can lead to severe or even life-threatening complications such as Purtscher-like retinopathy that can sometimes lead to blindness and hepatic subcapsular hematoma with a high-morbidity and -mortality rate, so that evaluation, monitoring, and therapeutic management of this condition should be multidisciplinary: obstetrician, surgeon, ophthalmologist, radiologist, and intensive care specialist to achieve favorable results.

## References

[1] Ferri FF (2012). *Ferri’s Clinical Advisor*.

[2] Coll J (2005). Hepatic subcapsular hematoma occurs in of 2% of HELLP syndrome complicated pregnancies. *College of Physicians and Surgeons Pakistan*.

[3] Stewart MW, Brazis PW, Guier CP, Thota SH, Wilson SD (2007). Purtscher-like retinopathy in a patient with HELLP syndrome. *American Journal of Ophthalmology*.

[4] Haddad B, Barton JR, Livingston JC, Chahine R, Sibai BM (2000). Risk factors for adverse maternal outcomes among women with HELLP (hemolysis, elevated liver enzymes, and low platelet count) syndrome. *American Journal of Obstetrics and Gynecology*.

[5] Haram K, Svendsen E, Abildgaard U (2009). The HELLP syndrome: clinical issues and management. A review. *BMC Pregnancy and Childbirth*.

[6] Kapana M, Evsenb MS, Gümüs M, Onder A, Tekbas G (2010). Liver hematoma in HELLP syndrome: case report. *Gastroenterology Research*.

[7] Nogales R, Vázquez L, Pereira I, Moreno C, White M (2007). López-Save A subcapsular hematoma hepatico, a de los Estados complicación infrecuente hypertensive del embarazo. *Clínica e Investigación en Ginecología y Obstetricia*.

[8] Carlson KL, Bader CL (2004). Ruptured subcapsular liver hematoma in pregnancy: a case report of nonsurgical management. *American Journal of Obstetrics and Gynecology*.

[9] Landes A, Jay WM (2009). Purtscher-like retinopathy in a patient with preeclampsia. *Seminars in Ophthalmology*.

[10] Murphy MA, Ayazifar M (2005). Permanent visual deficits secondary to the HELLP syndrome. *Journal of Neuro-Ophthalmology*.

[11] Agrawal A, McKibbin M (2007). Purtscher’s retinopathy: epidemiology, clinical features and outcome. *British Journal of Ophthalmology*.

